# Observations on Complicated Surgical Injuries, Including Gun-Shot and Other Wounds

**Published:** 1839

**Authors:** 


### OBSERVATIONS ON COMPLICATED SURGICAL INJURIES, INCLUDING GUN-SHOT AND OTHER WOUNDS. By *Rutherford Alcock*, Esq. late Deputy Inspector General of Hospitals with the Auxiliary Forces of Portugal and Spain.

MR. ALCOCK is a young surgeon of great attainments and high promise. He has served with distinction in the late campaigns in Portugal and Spain, and acquired an amount of experience in gun-shot and in other wounds, which, few in these piping times of peace can hope for. Nor has he suffered that experience to lie idle. He has been induced to communicate it to the profession, and to render it useful to us all.

The form of publication which has been adopted by Mr. Alcock has been that of lectures in our contemporary, the Medical Gazette. His observations have, in this manner, obtained an extensive circulation, and the information they contain has been conveniently diffused.

We shall select some of the more striking features in Mr. Alcock's remarks, and present them with little comment to our readers. We shall not attempt a connected analysis, as a lecture must necessarily contain much that is already familiar to practical men.

*Appearance of the Entrance and Exit of a Ball.*—There is more variety in the latter than in the former. Mr. A. has seen, however, in a wound of the face, the opening made by the entrance of the ball not only clear and incised, which is unusual, but so small that it was difficult to conceive the possibility of a ball having passed in. Generally the entrance of a ball is marked by a circularly depressed, torn, and contused opening, while the exit very commonly assumes more of the appearance of an incised wound—a sharp irregular line, frequently with everted edges.

*Erroneous Impressions of Patients.*—"It is often important to obtain clear ideas of the course a ball has taken; and in this matter the patient will sometimes entertain a very strong and erroneous impression. One of the most singular instances I remember was a soldier declaring he had spit out a ball which had lodged firmly in one of the cervical vertebræ, as you see by this preparation. On another occasion, an officer received a musket-shot in the flank, while turning to cheer a regiment on to the charge. I was by his side, and staggered at the same instant, under a violent shock caused, as I felt fully persuaded, by a ball through my knee. Having recovered in two or three moments from the first blinding and sickly feeling, I was well pleased to find that I had escaped with a grazing shot, and proceeded to see what was a matter with my companion, the Colonel. He had been carried a few yards down into a road, out of the shower of shot which had somewhat suddenly fallen upon us: thanks to the white breeches of the charging column. His mistake was a stranger one than my own; for when my forefinger was buried in the wound, following the track of the ball, he was giving himself no little trouble to convince me that the ball had not gone in, until I told him I thought it must, because—the point of my forefinger was four inches deep in his flank.

In fact, wounded men get all sorts of impressions, not only from balls, but about them. Nothing is more common than for a man to assure you he saw the ball drop after it struck him, or that he had picked it up, and persist in it, until, by the aid of such conjuring as a little judgment and a sharp knife will effect, the real Simon Pure is held to his eyes, still red and warm from his body."

Mr. Alcock is inclined to think that a number of slight shots are more dangerous, than one of a serious character.

*Extraction of Balls.*—"From many instances that I have seen of the great, and more than once fatal, anguish occasioned by the presence of foreign bodies, I am inclined to go further to insure their extraction than is generally recommended. If the ball can be felt, it matters not what depth of muscular parts may lie between, I would have it extracted, and, if necessary, incisions made for that purpose. Two cases more particularly impressed this upon my mind."

*Proportion of Cases of Secondary Hæmorrhage in the Wounded.*—Mr. Alcock thinks that he never had a batch of 300 wounded, without two, three, or more cases of secondary hæmorrhage.

In Portugal, in a set of 300, five cases occurred. In Spain, in one set of 400, five cases occurred: three in gun-shot fracture of femur; one in gun-shot wound of face; and one in gun-shot fracture of humerus.

In another set of 500, four cases: two from gun-shot fractures of bones of face, one of fibula, and one of radius into the elbow-joint. In 1350 cases under my charge at San Sebastian, nine: and in 197 officers, two cases—both gun-shot fractures of leg.

| | | | |
|---|---|---|---|
| 1350, rank and file, | 9 | cases of secondary hæmorrhage. | |
| 197, officers | - 2 | ditto. | |
| 1547 | 11 | | |

Mean average about 7 per 1000.

*Incomplete Division of an Artery does not necessarily produce Hæmorrhage.* Mr. Alcock observes that it is generally held, that an incomplete division of a large arterial trunk, whether by shot or incision, will prove instantaneously fatal if no effective means be taken to prevent it; occasionally, however, a very striking exception may occur. Mr. Alcock relates a very interesting case in illustration. He found a wounded man in a house, and examined him; he had apparently received a fatal wound in the face and neck. A musket-ball had entered the right cheek, on a level with the alveolar process of the last molar tooth, and passing downwards, made its exit in the neck of the opposite side, just above the clavicle, grazing the shoulder afterwards.

He lay totally insensible, labouring under the effects of concussion, with stertorous breathing, possibly also suffering from the loss of a large quantity of blood, although there was little about him where he was lying. Tumefaction ensued, suppuration, and on the fourth day the sloughs began to separate. On the fifth there was a little bleeding from both wounds—on the seventh it was considerable from the neck—on the eighth it occurred to the extent of two or three ounces—on the ninth, tenth, and eleventh, returns of it—on the twelfth, a violent hæmorrhage. On that day, Mr. Alcock tied the carotid, though great difficulties arose from the sloughy condition of the wound. The artery was exposed at a considerable depth from the surface, and a lacerated opening of nearly half an inch in extent was distinguishable in the canal of the carotid artery. The vein also appeared, though less distinctly, to be lacerated. The patient did well.

*Injuries of the Head.*

1. *Concussion.* Mr. Alcock defines this to be "any vibratory shock or jar, whether produced by direct violence to the head, or indirectly through any other part of the frame; producing lesion of function or structure in the brain."

It appears to us that we may apply the term "concussion" to one of two things;—*either* to a certain group of symptoms, more or less independent of their cause; *or* to a certain lesion, more or less independent of its symptoms. The latter is the more exact and philosophical method of proceeding, but it implies an acquaintance with the condition of the brain, in concussion, which unfortunately, we do not possess. The former plan then is the one actually adopted. If after an injury a patient exhibits certain cerebral symptoms, he is said to labour under concussion. If he dies, and *no inflammatory alteration or source of pressure* on the brain is found he is said to die of concussion. If he recovers without inflammatory symptoms, and without symptoms of pressure, he has recovered from concussion. If inflammatory symptoms supervene on those of concussion, with or without a perceptible interval, he is said to labour under inflammation of the brain or of its membranes. If symptoms of pressure come on then the case has merged in one of compression. Such is the usual phraseology, such are the current opinions. Mr. Alcock's definition would point to another, and we cannot help thinking a more vague notion of concussion, for "a shock producing lesion of structure in the brain" may include many injuries, which are not generally deemed mere concussion. We make this observation for the purpose of preventing any misconception in the minds of our readers, when they find Mr. Alcock employing a term in one sense which they might have understood in another.

Mr. Alcock remarks:—

"Stertorous breathing has been hitherto, and very erroneously, considered as a distinctive mark of compression, and not a symptom of concussion; but it occurs in both, and is merely indicative of a *degree* rather than a *kind* of injury. It indicates the implication of the excito-motory system—the relaxation of the sphincters—the stopping of the glottis. If the par vagum in addition has any thing to do with laboured breathing, the indication is the same as regards the implication of another system besides the cerebral.

Many cases of very severe lesion and compression occur without any stertor, the mischief or injury apparently being confined to the cerebral system. In a case of very extensive extravasation, tearing through a portion of the cerebrum, the details of which I will give hereafter, 'there was no stertor,' and the fact was thus distinctly marked during the patient's lifetime. In another case of pure ramollissement, to which I shall also refer later, there was distinct and loud stertor for many hours.

Thus neither does the presence of stertor certainly indicate compression, nor its absence prove that no such state exists. I repeat it is an evidence of degree when the injury is in the brain, rather than of kind, and may occur in all forms."

Nothing can be more just than the observation that stertor is not a necessary symptom of compression, nor a certain proof of it's existence. All physicians are aware that ramollisement will produce all the usual symptoms of pressure—and many surgeons are not ignorant that pressure will produce convulsions or delirium, without stertor or the usual indications of pressure.

The real difficulty of distinguishing concussion from compression hinges on our ignorance of the pathological condition of which concussion is the symbol. We are therefore, as we said before, obliged to erect a mere group of symptoms into a substantive affection. But these symptoms may depend on more than one morbid condition of the brain, and the difference between that and compression may sometimes be one of kind and sometimes one only of degree. Hence the necessity for extreme caution in defining these several lesions, and in erecting theoretical barriers between affections, the precise distinctive characters of which are not well known.

Mr. Alcock observes:—

"The most invariable consequence of all severe injuries to the head is concussion. I conceive it of the greatest importance to define in what this consists, and what are the effects which may be truly traced to it. The four elements of head injuries I have stated are, concussion, compression, lesion, ramollissement; and any or all of the three latter may be effects of the first. In this order they are best considered. Earliest in order of these effects is, a frequently recurring coma, supervening an hour or two after a concussion, though not at first, without any perceptible compression or lesion of structure otherwise to account for it.

'A question suggests itself—

'May not concussion lead to a subsequent comatose state, not depending on pressure or lesion, or any perceptible alteration of fibre or structure, an effect of concussion simply and only?'

The frequency of a comatose state supervening shortly after the shock, but not at the time, gave me this impression; but on careful investigation I failed in satisfying myself of the soundness of such a conclusion; but I succeeded in assuring myself, that the drowsiness, lethargy, or stupor, thus supervening, was *not* the result of compression, as that term is usually understood.

These cases do not admit of explanation by occular demonstration, for either the patient gradually recovers, leaving us in doubt whether he recovers from mere concussion, or by the absorption of matter or other fluid which might have caused pressure; or if, on the contrary, he dies, there is such extensive disorganisation or mischief, that the post-mortem evidence still fails to decide the question.

Close observation, and the fair use of our reasoning faculties, will often surmount greater difficulties than these. Attention to many such cases showed me, before long, that this supervening comatose state certainly did not arise from any pressure by effused fluid or coagulum, still less by formation of matter; first, because it came on too suddenly; and secondly, it might by treatment be as promptly dispelled. Absorption of any matter effused, secreted, or extravasated could not possibly be the agent or mode of relief.

I conceive this effect may arise from the pressure of turgid and over-distended vessels throughout the substance of the brain—and the prompt relief, although often only temporary, produced by copious bleeding, further confirmed me in this opinion—or otherwise, the fibre may be so debilitated in its power of resistance, that it will not bear the pressure of even the normal quantity of blood in its substance: in either case it would be a case of compression; but compression not as it is usually understood or defined, nor possibly even congestion.

The following case bears upon this point:—

CASE, *showing the Secondary Coma of Concussion by pressure from fluid within the vessels.*—Philip Thorne, æt. 24, on the 16th of March, 1837, walked into the hospital from the field, and conversed intelligently for some time after he was placed in the ward; he had been struck by a musket-ball on the os frontis, which was fractured. Soon after he became quite comatose. Pulse slow and weak; surface pale and benumbed. His head was shaved; cold applied. About nine in the evening a small arterial branch bled very freely, which speedily relieved the appearances of compression, and the next morning he was free from all bad symptoms.

Spoon diet. Sharp purgative of calomel and jalap administered.

3rd day there was a little pain the head; pulse slow.

4th.—He said he felt occasionally light-headed; pulse rather jerking, but easily compressed; secretions free from the bowels.

5th—No change.

6th—Unusually sluggish and languid pulse, 56; complains only of local pains; sore unhealthy; bone denuded beneath.

7th—Pulse 52, same character.

8th.—Right pupil slightly dilated; pulse 48, soft and irregular; very little pain, and merely local. During this period a saline mixture cum ant. tart, was directed for him.

9th—Says he feels perfectly well; all natural but pulse, which, up to the 15th, remained at 52, during which time he continued rapidly improving. It then became 70, regular, and perfectly natural.

He was discharged on the fourth month perfectly well, save that exposure to the sun, or much exertion, brought on pain and giddiness in the head.

This case then shows that a blow of no common violence did not produce any loss of consciousness, or serious inconvenience in the first instance; that in a few hours complete coma supervened, and was almost instantly relieved by loss of blood; the same state in a lesser degree, returning the second day after, and the pulse continuing to the fifteenth day to indicate disturbance of the circulation, but in no other organ or division of the nervous system.

It would be easy to select many cases where stupor supervened, sometimes after concussion and not at first, as readily relieved by bleeding, returning again, and that two or three times in the treatment of the same case; but as they are cases complicated with lesion of brain, &c., they give less simple, and therefore, perhaps, less conclusive evidence on the particular points under discussion. This tardy or 'secondary comatose condition,' as I have termed it, quickly relieved by bleeding, again recurring and again relieved, it must be evident, is the result of the shock, the concussion. It does not occur by any extravasation of fluid; and if from pressure, apparently can only arise from pressure of the fluid in the blood-vessels, whether in ordinary or extraordinary quantity."

There cannot, we think, be much hesitation in subscribing to Mr. Alcock's opinions. The fair and usual case of "concussion" is where the symptoms follow hard upon the injury—this "secondary coma" must, in the present condition of our knowledge, be looked on as the result of congestion, which, if not checked by depletion, would, probably, in many cases, run on to inflammation. It certainly should be distinguished from the common and simpler case of concussion, both on account of its nature and its treatment. It approaches, in our minds, more to compression than concussion. It must be recollected that some of the worst cases of apoplexy are, apparently, of a similar description. A man dies with marked apoplectic symptoms. On dissection, all that is found is a congested condition of the brain. A blow on the head would be likely, à priori, to give rise to such a state, and even were other evidence wanting, the case and the observations of Mr. Alcock, would, to our minds, be perfectly conclusive.

Mr. Alcock proceeds to observe that cases, apparently similar, will end in very different ways. If the lesions are really similar, the difference of termination seems to us to be *usually* attributable to the variety in the degree or kind of succeeding inflammation. This is after all no more than we see in all accidents of all parts, and indeed in all instances of all morbid actions.

Mr. Alcock next alludes to what he terms "the dispersed or distant effects" of injuries of the head upon the system. These, however, are no other than those secondary inflammations and deposites, which have attracted great attention of late years, and which follow other injuries as well as those under consideration, and we are much inclined to doubt whether such inflammations and deposites can be looked upon as the effects of concussion. They follow those injuries of the head, which lead to inflammation and to suppuration, or the formation of lymph. There is every reason to believe that it is to the latter, and not to the concussion, per se, they are attributable. At all events, we have never seen them result from mere concussion, and we have seen them ensue after scalp-wounds and injuries unattended with concussion altogether.

Mr. Alcock pursues the effects of concussion.

"It produces," he says, "an irritability of fibre, rendering the brain morbidly sensitive to all stimulus; and this, I am led to believe, is by far the most common effects of serious concussion. Any excitement—much exertion of mind—exposure to the rays of the sun—spirituous liquors, &c., quickly producing head-ache and giddiness: if persisted in or carried to excess, violent disease in some form is developed in the brain. I have almost invariably found this to be one of the effects, more or less permanent, of serious concussion, and often the only one remaining after treatment."

Mr. Alcock relates two cases. We will quote the second.

*Case.*—William Wilson, æt. 34, was struck by a ball on the outer angle of the forehead on the left side, and the bone was slightly fractured. There do not appear to have been any of the usual symptoms of concussion.

Second day, a little pain of head; third, easy; no fever; tranquil pulse.

On the fourth day complained of a great deal of pain and uneasiness about the head.

A sharp purgative of cal. and colocynth, followed by magn. sulph. and ant. tart.

On the succeeding day was found to have passed a restless night; eyes heavy; pupils dilated; pulse slow, laboured, and full in volume. Bowels had been freely purged.

V. S. ad ℥xvj. Emp. lyttæ nuchæ.

6th day.—Little change; no sleep, but feels heavy; pupils dilated, and vision of left eye (below the wound) impaired.

7th.—Improved a little; less heaviness of head; bowels open; pulse calm and soft; tongue clean; thirst; pain in the head still; eyes suffused.

Repeat the blister.

8th.—Pulse more sluggish. Pain continues.

9th.—Better; less pain and heaviness of head; pupils contract slowly; redness of eyes continues; pulse soft, and nearly natural.

10th.—Upon the whole feels better. Pupil of left eye widely dilated—less redness. Pulse soft and tranquil; tonge moist and clean; skin cool; extremities warm.

From this period to the end of the third month, pain of the head, conjunctival disease, sluggish action of bowels, and impaired vision of left eye, continued. He was at this period sent home, when Mr. Alcock made the following note of his state.

" Dec. 31st.—Invalided home. He complains of constant pain in the head, more or less severe. Injury to the sight of the left eye—a sparkling before it. Pain shooting from the point where he was wounded, across the forehead; giddiness. Both pupils appear more dilated than natural. No other indication of disease, or variation from healthy state. His tongue red, clean, and appetite good. Pulse 92, full, and regular. A trifling cicatrix marks where the ball struck and apparently glanced off.

This case is an example of a high degree of irritability of fibre, and to this I refer the almost unceasing and long-continued pain of the head, without any other indication of disturbed health. Here no unusual stimulus was required to produce the headache, &c."

It appears to us that, for the sake of precision both of language and ideas, it is always advisable to connect, so far as we are able, effects with their immediate causes. The preceding case did not display, so far as we can see, the symptoms which are termed concussion. On the other hand, the subsequent phenomena, the pain in the head, the suffusion of the eye, the loss of vision on the side corresponding to the blow, rather tend to create a supposition that some inflammatory, or, at all events, congestive action was set up in or about the brain. We do not deny that a peculiar state of excitability is sometimes left after pure concussion, not succeeded by inflammatory action, or not attended by some obvious lesion. Yet it strikes us that such cases are not so frequent as those where a distinct lesion or positive vascular action plays the efficient part.

If a blow on the head produces no symptoms of " concussion" at the time, and, after an interval coma supervenes—is that to be looked on as concussion? The question is rather one of words than things; but so far as we have seen, such coma has been attributable to congestion or incipient inflammation of the encephalon, marked by characters which were tolerably appreciable. " Concussion," or nervous shock, is not usually benefitted by depletion—this supervening coma is.

However this may be, or whatever view of the subject may be taken, we proceed with Mr. Alcock's observations.

"Continuing," he says, "the classification of the effects of concussion, there sometimes will result inflammatory or mixed inflammatory and irritable symptoms, without any intervening coma, stupor, or loss of consciousness, of which I will give you an example. Let me observe, first, that this effect collaterally proves the fact I have assumed, that the secondary coma (*i. e.* a violent effect) may proceed from irritability, a state of cerebral fibre induced by concussion; although that concussion may not, in the first instance, have produced lesion of functions, organic or animal."

In other words, concussion may be followed by inflammatory action in the brain or its membranes.

Another effect of concussion is thus noticed by Mr. Alcock:—

In some cases, during the first comatose stage or continuance of coma, particularly if the concussion be complicated by any lesion or lodgement of a foreign body, an exalted action supervenes, and inflammation or irritability becomes developed. The pupil contracts and the pulse beats sharply.

Mr. Alcock gives, as an instance, a case in which a man received a musket-ball, which penetrated the scalp, over the centre of the right parietal bone, fracturing and depressing a portion. The dura mater was torn—portions of bone were forced into the substance of the posterior part of the right hemisphere—the ball, jagged and flattened on one side, was lodged in the brain, half an inch from its surface—the structure of the brain was ulcerated and destroyed nearly perpendicularly downwards to the roof of the right lateral ventricle. The whole of the right was exceedingly softened; the left firm and natural. No obvious traces of inflammation in the structure generally. Right lateral ventricle filled with turbid fluid, and the whole surface somewhat discoloured, and apparently implicated in the disease. The whole of the dura mater, at the base, was of a bright purple colour. Right cerebellum slightly softened. The patient was comatose on admission, with contracted pupils, and full and bounding pulse. He recovered partially from the coma. He died on the 27th day.

Mr. Alcock refers another class of effects to concussion:—little loss of consciousness or coma at first, but quickly succeeded by disorganization of the brain, partial or general.

He cites the following short case.

*Case.*—John Medley, was struck on the first of October, 1836, by a musket-ball, which entered the back of the head at the left side, coursed below the integuments, and was cut out over the parotid gland of right side. The constitutional or sensorial disturbance very slight, and at eight in the evening he was in an easy sleep.

On the 2nd day he was drowsy, and rather inclined to wander; pulse was feeble, and very slow.

V.S. ad ℥xvj. Cold applications to the head.

3d.—Moribund; and on the 4th he died.

*Post-mortem.*—The ball was found to have struck the bone below the occipital protuberance, to have driven in a splinter on the dura mater, then passed on externally, and lodged under and to the inside of the mastoid muscle. The dura mater was slightly torn. The brain for some distance about the wound was dark, sloughy, and disorganised; all the vessels were injected, and the ventricles full of serum. The ball had not fractured the skull where it pierced the scalp, but obliquely in its passage.

Nothing can exhibit in a stronger light than this case does, the different sense attached by Mr. Alcock, and by surgeons generally, to the term concussion. Most persons would say that in this instance the concussion was slight, and the lesion of the brain by the splinter the essential cause of it's disorganization and the patient's death.

It is only fair, however, to our able author, to cite his own explanation of his sentiments.

"To successfully distinguish between compression and concussion when both have taken place, is not always possible; and I think the endeavour has often led to error. It has led to the practice of considering, that if after a comatose state, from concussion, or even without that effect having occurred, if some hours or days after, a state of stupor, &c. come on, the surgeon in such case had undubitably to deal with compression, and that only. It will be seen by the facts I have already brought forward, that such can rarely if ever be the case; that the effects of concussion do not cease with even the first stupor, but that there are manifold effects assuming many forms, which follow as natural sequences. It is exceedingly rare that some one or other of these do not ensue; and if compression be one of them, it is not compression only that baffles our skill or taxes our diagnostic powers, but compression added to concussion, from which in truth it proceeds. I have endeavoured to show that this very compression may exist by the blood within the vessels, and only by the altered fibre of the brain, induced by concussion. Nay, if lesion, disorganization, and ulceration ensue, we still are among the sequelæ of concussion, when that has existed in the first instance; and if to concussion they add some features or symptoms, which yet remain to be seen, still they are enveloped, pervaded, as it were, by the general characteristics, and controlled by the injurious influences of concussion. I have yet to show that concussion, by its disorganising effects will *control the outward manifestations of inflammation, and maintain the action of the heart at its own lowered standard.* It will paralyze and rigidly fix the iris, in despite of inflamed membranes and brain, the natural effect of which is greatly to increase the irritability of the retina, and endue the iris with morbid contractility. It will permit the development of inflammation within the skull, and yet control the manifestation of any increased action throughout the system; where, then, are the unerring guides to be sought for on the supervention of inflammation, lesion, or extravasation? Not in the pulse, not in the iris, neither in the skin; all these it will control at periods, when it has hitherto been deemed to have ceased all action. Without concussion or a blow, compression may often be truly predicated; lesion I doubt if ever, and not always compression; for I have also shown that disorganization and softening will produce symptoms undistinguishable from concussion and compression."

Mr. Alcock sums up the usual stages or phases of concussion thus:—

1. Coma, depressed or paralysed action, which may be confined to cerebral system, or implicate in various degrees the excito-motory and the sympathetic.

2. Exalted action more or less extended in a similar manner, and varying in degree in each; attended almost invariably with suppressed or diminished secretions.

3. Stage marked by irritability of cerebral fibre existing in some for days, and often for life.

We cannot conclude without expressing the sense we entertain of the zeal and the abilities of Mr. Alcock. He distinguished himself honourably in the Peninsula, and is a candidate (we have no doubt he will be a successful one) for metropolitan practice.

